# Occupational and environmental iron exposure: a hidden risk factor in breast cancer etiology

**DOI:** 10.1097/MS9.0000000000004305

**Published:** 2025-11-24

**Authors:** Emmanuel Ifeanyi Obeagu

**Affiliations:** Department of Biomedical and Laboratory Science, Africa University, Mutare, Zimbabwe

**Keywords:** breast cancer, environmental pollution, iron overload, occupational exposure, oxidative stress

## Abstract

Breast cancer remains a major public health challenge globally, with its etiology influenced by a complex interplay of genetic, hormonal, and environmental factors. While traditional risk factors such as age, reproductive history, and family predisposition are well-recognized, emerging evidence points to heavy metal exposure – particularly iron – as a potential contributor to carcinogenesis. Iron is a vital micronutrient required for cellular metabolism; however, excess iron has been shown to catalyze oxidative stress and deoxyribonucleic acid damage, both of which are implicated in tumor initiation and progression. Occupational and environmental iron exposure is increasingly prevalent due to industrial expansion, especially in metal-processing industries such as welding, mining, and steel manufacturing. Workers in these sectors, as well as individuals living near industrial zones, may experience chronic low-level exposure to iron through inhalation, ingestion, or dermal absorption. When iron accumulates beyond the body’s regulatory capacity, it may foster a pro-oxidative and pro-inflammatory milieu conducive to breast tumorigenesis. Furthermore, iron’s interaction with estrogen metabolism may have unique implications for hormonally sensitive tissues like the breast.

## Introduction

Breast cancer is the most frequently diagnosed malignancy among women worldwide and remains a leading cause of cancer-related mortality^[1]^. Despite significant advances in detection and treatment, the incidence continues to rise, particularly in developing countries undergoing rapid industrialization and urbanization^[2]^. Breast cancer etiology is multifactorial, with well-established risk factors including genetics, hormonal influences, reproductive history, lifestyle behaviors, and environmental exposures^[3]^. However, an increasing body of evidence suggests that exposure to certain metals, including iron, may contribute to the carcinogenic process, warranting closer examination^[4]^. Iron is an essential trace element that plays a pivotal role in numerous biological processes such as oxygen transport, deoxyribonucleic acid (DNA) synthesis, and cellular respiration. The human body maintains iron homeostasis through tightly regulated mechanisms of absorption, storage, and recycling. Despite its necessity, excess iron can be highly toxic due to its ability to catalyze the formation of reactive oxygen species (ROS) via the iron-catalyzed reaction, producing ROS (Fenton reaction). These ROS can induce oxidative damage to DNA, lipids, and proteins, potentially initiating or promoting carcinogenesis^[5,6]^. Thus, while iron deficiency has been widely studied, the harmful effects of iron overload in the context of cancer risk have received comparatively less attention.

**HIGHLIGHTS**

- Iron exposure promotes oxidative stress, DNA damage, and carcinogenesis.
- Occupational settings increase women’s cumulative iron load.
- Environmental iron pollutants contribute to breast tissue alterations.
- Iron dysregulates estrogen metabolism.
- Early detection requires metal exposure screening.

Occupational and environmental sources represent significant but often overlooked contributors to iron overload in humans. Industrial activities such as mining, metal processing, welding, and steel manufacturing generate iron-containing particulates that workers can inhale or absorb through the skin. Moreover, environmental contamination from industrial emissions and waste disposal can elevate iron levels in air, water, and soil, exposing nearby populations to chronic low-level iron intake^[7]^. Such exposures may be particularly relevant in regions with lax environmental regulati^]^.ons or poor occupational safety standards. The breast tissue is hormonally sensitive and vulnerable to oxidative stress-induced damage^[8]^. Excess iron may exacerbate this vulnerability by promoting oxidative DNA damage and influencing estrogen metabolism, potentially leading to mutagenesis and tumor initiation^[9]^. Inflammatory processes induced by iron overload can further create a microenvironment conducive to tumor progression^[10]^. Given that breast cancer development involves both genetic and environmental factors, understanding the role of iron exposure adds an important dimension to unraveling its complex etiology.

Although studies have linked systemic iron overload syndromes, such as hereditary hemochromatosis (HH), to increased cancer risks, the specific impact of chronic occupational and environmental iron exposure on breast cancer remains underexplored. Epidemiological investigations have been limited and often inconclusive, partly due to challenges in accurately assessing individual exposure and differentiating iron’s effects from those of other metals or carcinogens^[11]^. Nonetheless, growing experimental and observational evidence suggests that iron’s carcinogenic potential should not be ignored^[12]^. This review aims to synthesize current knowledge on the biological mechanisms through which excess iron may contribute to breast carcinogenesis and to examine the evidence linking occupational and environmental iron exposure to breast cancer risk.

## Aim and specific objectives

### Overall aim

The primary aim of this review is to comprehensively evaluate the relationship between occupational and environmental iron exposure and the risk of breast cancer, integrating epidemiological evidence, mechanistic insights, and public health perspectives. The review seeks to uncover how excess or chronic iron exposure – whether through industrial, dietary, or environmental sources – may contribute to breast carcinogenesis, disease progression, and population-level risk disparities.

### Specific objectives

1. **To synthesize quantitative epidemiological evidence**

This objective focuses on consolidating data from recent cohort, case–control, and population-based studies that assess the association between iron exposure and breast cancer risk. Emphasis is placed on presenting quantitative measures such as relative risk (RR), odds ratio (OR), and 95% confidence intervals (CI) to evaluate the strength and consistency of reported associations across different populations and exposure settings.

2. **To explore biological and molecular mechanisms linking iron overload to breast tumorigenesis**

The review examines how dysregulated iron metabolism contributes to oxidative stress, DNA damage, lipid peroxidation, and ferroptosis, ultimately fostering tumor initiation and progression. It also analyzes the role of key molecular mediators – such as hepcidin, ferritin, and transferrin receptor – in regulating intracellular iron and influencing oncogenic signaling pathways, including nuclear factor kappa-light-chain-enhancer of activated B cells (NF-κB), HIF-1α, and PI3K/AKT.

3. **To assess occupational and environmental sources of iron exposure and their implications for women’s health**

This objective identifies high-risk exposure pathways such as metal industry emissions, mining activities, contaminated water or soil, and dietary iron excess. It further discusses the influence of occupation, residence, and environmental inequities on exposure levels, particularly in developing regions where monitoring and regulation are limited.

4. **To evaluate the potential role of iron as a biomarker and modifiable risk factor in breast cancer prevention**

The review explores whether serum ferritin, transferrin saturation, and total iron-binding capacity can serve as early biomarkers of exposure or disease risk. It also discusses the potential for preventive interventions – such as iron chelation, dietary modification, and environmental control measures – to reduce exposure-related breast cancer risks.

5. **To propose public health and policy recommendations**

This objective outlines evidence-based strategies for improving occupational safety, strengthening environmental regulations, and integrating iron exposure assessment into breast cancer prevention frameworks. It emphasizes the importance of surveillance, risk communication, and intersectoral collaboration to mitigate iron-related cancer risks.

## Methods

This narrative review was conducted to synthesize current evidence on the relationship between occupational and environmental iron exposure and the risk of breast cancer. The methodological framework followed a structured yet flexible approach suitable for narrative reviews, emphasizing breadth of coverage and critical interpretation of existing data.

### Search strategy

A comprehensive literature search was performed across four major databases – PubMed, Scopus, Web of Science, and Google Scholar – to identify relevant studies published between January 2000 and July 2024. The search used combinations of controlled vocabulary and free-text terms, including *iron exposure, environmental iron, occupational iron, breast cancer, ferritin, transferrin saturation, oxidative stress*, and *ferroptosis*. Boolean operators (AND, OR) were employed to refine the search and ensure inclusion of all relevant materials.

### Inclusion and exclusion criteria

Studies were included if they

1. Were peer-reviewed and published in English;
2. Investigated human populations with either occupational or environmental exposure to iron;
3. Reported breast cancer incidence, risk, or mechanistic correlations; and
4. Included either quantitative or qualitative data relevant to exposure–outcome relationships.

Excluded materials comprised animal-only studies lacking translational context, case reports, editorials, commentaries, and conference abstracts without full data. Review papers were consulted for citation tracking and background synthesis but were not used as primary data sources.

### Data extraction and synthesis

Two authors independently screened titles and abstracts for eligibility, followed by full-text review. Disagreements were resolved through consensus. Extracted data included study type, population characteristics, sample size, type and source of iron exposure, biomarkers used (e.g., ferritin, transferrin saturation), outcome measures, and statistical indicators such as ORs, RRs, or hazard ratios (HRs) with 95% CIs.

### Quality appraisal

Although formal risk-of-bias tools were not applied due to the narrative nature of this review, each included study was critically assessed for

- Appropriateness of study design (e.g., cohort vs. case–control),
- Accuracy and validity of exposure and outcome measure-ments,
- Statistical adjustment for potential confounders, and
- Clarity in reporting results.

Studies with clear definitions of iron exposure and breast cancer outcomes, adequate sample size, and rigorous statistical analyses were considered higher-quality evidence.

### Presentation of findings

Findings were organized into thematic domains reflecting the main objectives of the review:

1. **Epidemiological associations** between iron exposure and breast cancer risk;
2. **Molecular and mechanistic pathways** (oxidative stress, ferroptosis, hormonal dysregulation, and inflammation); and
3. **Public health and preventive perspectives** related to occupational safety, environmental monitoring, and iron modulation strategies.

### Quantitative evidence linking iron exposure and breast cancer risk

Quantitative epidemiological data increasingly support an association between elevated iron exposure or body iron stores and increased breast cancer risk. Case–control investigations have reported markedly higher odds of breast cancer in women with elevated serum ferritin and transferrin saturation; for example, Von Holle *et al*^[13]^ observed an OR of 2.46 (95% CI 1.38–4.38) for women with high ferritin compared with lower levels, with correlations to tumor grade and stage. Similar case–control evidence from an East Asian population found that women in the highest quartile of serum iron had increased odds of breast cancer (OR = 1.78; 95% CI 1.22–2.59)^[14]^. Prospective cohort data likewise indicate modest but consistent elevations in risk: in the Women’s Health Initiative cohort, participants in the upper plasma ferritin quartile had an HR of 1.25 (95% CI 1.03–1.52) for incident breast cancer, suggesting that higher systemic iron stores predate disease onset^[15]^. Dietary and environmental exposure studies complement these biomarker findings: a prospective Chinese cohort reported increased risk of estrogen receptor–positive breast cancer with higher dietary heme iron intake (RR = 1.41; 95% CI 1.10–1.82), and a Taiwanese population-based cohort linked long-term exposure to iron-contaminated groundwater with an elevated hazard of breast cancer (HR = 1.32; 95% CI 1.06–1.63)^[16]^. Additionally, analyses of supplemental and total iron intake in multiethnic cohorts have suggested modestly increased risks in postmenopausal women (RR = 1.30; 95% CI 1.05–1.62)^[17]^.

While these estimates – ORs generally in the 1.3–2.5 range and HRs up to ~1.3 – are consistent across diverse designs and populations, important caveats apply. Study heterogeneity in exposure assessment (serum biomarkers, dietary recall, environmental measures), residual confounding (coexposure to other metals, hormonal and reproductive factors, obesity), and variable adjustment sets limit causal inference. Nevertheless, the convergence of biomarker, dietary, and environmental findings strengthens the plausibility that iron overload functions as a cocarcinogen in breast tissue via oxidative, hormonal, and inflammatory mechanisms. We therefore recommend that future large-scale prospective studies include standardized iron exposure metrics and rigorous confounder control to better quantify dose–response relationships and identify susceptible subgroups^[18]^.

### Biological importance and homeostasis of iron

Iron is an indispensable micronutrient essential for numerous physiological processes across virtually all living organisms. It functions as a critical component of hemoglobin, enabling oxygen transport from the lungs to peripheral tissues. Additionally, iron serves as a cofactor for various enzymes involved in cellular respiration, DNA synthesis, and metabolic pathways. These vital roles underscore why iron deficiency can lead to anemia, impaired immunity, and reduced cognitive function. Conversely, the body’s iron levels must be tightly regulated because of iron’s potential toxicity when in excess. The homeostatic control of iron balance is primarily maintained through regulation of dietary absorption, as the body has no physiological mechanism for active iron excretion. Enterocytes in the duodenum carefully regulate iron uptake based on systemic iron demands, modulated by the hormone hepcidin, which acts as a master regulator. Hepcidin inhibits iron export by binding to ferroportin, the only known cellular iron exporter, thereby reducing iron absorption and release from macrophages. This feedback loop ensures iron availability while preventing overload^[19]^. Systemic iron is transported in plasma bound to transferrin and stored intracellularly mainly as ferritin, a protein complex that sequesters iron in a nontoxic, bioavailable form.

Excess iron, especially in its free or labile form, catalyzes the generation of ROS through the Fenton and Haber–Weiss reactions. These ROS can induce oxidative damage to nucleic acids, lipids, and proteins, triggering cellular dysfunction, mutation accumulation, and apoptosis dysregulation – processes intimately involved in carcinogenesis^[6,20]^. Therefore, iron’s biological duality as both a life-sustaining nutrient and a potential pro-oxidant highlights the importance of maintaining its balance. In the context of breast tissue, the tight regulation of iron is crucial, as epithelial cells are particularly sensitive to oxidative stress and DNA damage. Disruptions in iron homeostasis can sensitize breast cells to oncogenic transformation, especially in conjunction with other carcinogenic insults^[21]^. Understanding iron’s physiological roles and the mechanisms underlying its regulation is foundational to appreciating how occupational and environmental exposures might contribute to breast cancer risk via iron overload.

### Sources of occupational and environmental iron exposure

Iron exposure in human populations occurs not only through dietary intake but also via occupational and environmental pathways, which can lead to excessive accumulation beyond physiological needs. Understanding these sources is critical to assessing risk and implementing preventive measures, particularly in vulnerable populations.

#### Occupational exposure

Occupational settings are a primary source of elevated iron exposure, especially in industries involving metal extraction, processing, and fabrication. Workers in mining, smelting, welding, foundries, steel manufacturing, and construction are frequently exposed to iron-containing dust, fumes, and particulates. Inhalation of iron oxide particles during welding and grinding activities is particularly notable, as these fine particles can penetrate deep into the respiratory tract, leading to systemic absorption^[22,23]^. Prolonged exposure in poorly ventilated environments or where personal protective equipment is inadequate increases the risk of iron accumulation and associated oxidative stress. Additionally, dermal exposure through contact with iron-laden materials and accidental ingestion of contaminated dust may contribute to elevated body iron levels. Occupational iron exposure is often chronic and cumulative, reflecting the extended duration many workers spend in these environments. Despite occupational safety regulations in some regions, inconsistent enforcement and lack of awareness result in ongoing risks, particularly in low- and middle-income countries where industrial growth may outpace regulatory development^[24,25]^.

#### Environmental exposure

Environmental iron exposure arises from natural sources and anthropogenic activities. Naturally, iron is abundant in soil and groundwater; however, industrial pollution significantly elevates environmental iron concentrations^[26]^. Emissions from iron and steel plants, mining operations, and fossil fuel combustion release particulate matter containing iron into the air. Populations residing near industrial complexes or in urban areas with heavy traffic are consequently exposed to higher levels of airborne iron particulates, which can be inhaled or deposited on crops and water sources^[27,28]^. Water contamination with iron occurs via leaching from industrial waste, mining effluents, and corroded pipes, leading to ingestion of elevated iron levels. Although iron in drinking water is generally not acutely toxic, chronic exposure to excessive iron through this route may contribute to systemic overload, particularly in sensitive individuals^[29]^. Soil contamination also affects agricultural products, potentially introducing iron into the food chain beyond dietary norms^[30]^.

### Mechanisms linking iron exposure to breast carcinogenesis

Iron is an essential trace element involved in oxygen transport, DNA synthesis, and cellular metabolism. However, chronic or excessive iron exposure – whether occupational, environmental, or dietary – can disrupt cellular homeostasis and promote carcinogenesis in breast tissue through several interconnected mechanisms^[31,32]^. Iron catalyzes the formation of ROS via Fenton and Haber–Weiss reactions, producing highly reactive hydroxyl radicals capable of damaging DNA, proteins, and lipids. Oxidative DNA lesions, including 8-oxo-2’-deoxyguanosine (8-oxo-dG), can result in mutations within oncogenes and tumor suppressor genes, initiating tumorigenesis. Elevated iron stores, as measured by serum ferritin or transferrin saturation, have been correlated with increased oxidative stress biomarkers in breast tissue, supporting a mechanistic link between iron overload and genomic instability^[33,34]^.

Ferroptosis, an iron-dependent form of regulated cell death characterized by lipid peroxidation, plays a dual role in breast cancer. While ferroptosis can eliminate damaged cells under normal circumstances, chronic iron overload may trigger adaptive responses in malignant cells, enabling survival in oxidative environments. Key regulators such as glutathione peroxidase 4 (GPX4), SLC7A11, and ferritin heavy chain (FTH1) modulate ferroptotic sensitivity. Dysregulation of ferroptosis pathways can therefore facilitate tumor progression and resistance to therapy^[29,30]^. Iron can influence estrogen metabolism through the redox cycling of catechol estrogens, producing DNA-damaging quinones. These estrogen-derived metabolites interact with iron to generate ROS and form DNA adducts, particularly in estrogen receptor–positive breast cancer cells. By amplifying oxidative DNA damage in hormonally sensitive tissues, iron contributes to the initiation and promotion of breast tumorigenesis^[33–36]^.

Iron accumulation in macrophages and other immune cells can provoke the release of pro-inflammatory cytokines, including interleukin-6 (IL-6) and tumor necrosis factor-alpha (TNF-α). This inflammatory milieu promotes angiogenesis, immune evasion, and extracellular matrix remodeling, creating a microenvironment conducive to tumor growth and invasion. Chronic iron-induced inflammation, therefore, links environmental and occupational exposure to breast cancer progression^[37,38]^. Excess iron can also induce epigenetic alterations, including DNA methylation changes and histone modifications, which affect the expression of oncogenes and tumor suppressor genes. Iron-mediated ROS influence key signaling pathways such as NF-κB, HIF-1α, and PI3K/AKT, further enhancing cell proliferation, survival, and metastatic potential. These molecular perturbations provide a mechanistic basis for the observed epidemiological associations between iron exposure and breast cancer risk (Table 1) ^[39]^.

**Table 1:** Mechanistic pathways linking iron exposure to breast carcinogenesis

Pathway	Description	Key molecular players	Impact on breast cancer
Oxidative stress and DNA damage	Excess iron catalyzes Fenton reactions → ROS generation → DNA oxidation	Fe^2^⁺/Fe^3^⁺, •OH radicals, 8-oxo-dG	Induces mutations in oncogenes/tumor suppressor genes
Ferroptosis dysregulation	Iron-dependent lipid peroxidation → regulated cell death or survival adaptation	GPX4, SLC7A11, FTH1	Can promote tumor survival under oxidative stress
Hormonal modulation	Iron interacts with catechol estrogens → redox cycling → DNA adduct formation	Estradiol metabolites, catechol estrogens	Enhances ER-positive breast cancer risk
Inflammatory microenvironment	Iron accumulation → macrophage activation → cytokine release	IL-6, TNF-α, ferritin	Promotes angiogenesis, immune evasion, and tumor invasion
Epigenetic modifications	Iron-induced ROS alters methylation and histone modifications	DNMTs, histone demethylases	Silences tumor suppressor genes, upregulates oncogenes

### Epidemiological evidence

The epidemiological association between iron exposure and breast cancer risk remains an evolving field with growing but still limited direct evidence. Most studies addressing this link have focused on systemic iron overload conditions or indirect measures of iron status, while research specifically targeting occupational and environmental iron exposures and breast cancer is comparatively sparse^[40]^.

#### Iron status and breast cancer risk

Several observational studies have examined serum iron markers, such as ferritin, transferrin saturation, and total iron-binding capacity, in breast cancer patients compared to healthy controls. Elevated serum ferritin levels – a surrogate marker of body iron stores – have been frequently reported among breast cancer cases, suggesting a correlation between systemic iron overload and increased cancer risk. However, the causal direction remains to be definitively established, as ferritin is also an acute-phase reactant that may rise with inflammation. Nonetheless, these findings support the hypothesis that excess iron contributes to carcinogenic processes^[39]^.

#### Occupational exposure studies

Epidemiological investigations involving workers exposed to iron-containing dust and fumes provide indirect insights. Studies of welders, miners, and metal workers have documented higher overall cancer incidence and mortality, with some evidence of increased risks for respiratory and digestive tract cancers. However, data specifically linking occupational iron exposure to breast cancer incidence are limited and often confounded by coexposure to other carcinogens such as asbestos, silica, or polycyclic aromatic hydrocarbons. A few case–control and cohort studies in industrialized regions have noted elevated breast cancer rates in women employed in metal-processing industries, though these associations require confirmation through larger, methodologically rigorous studies. Accurate exposure assessment and differentiation between various metal exposures remain significant challenges in these investigations^[13]^.

#### Environmental exposure and geographic correlations

Ecological and geographic studies have also examined breast cancer incidence in relation to environmental metal pollution, including iron. Areas with high industrial activity or iron-rich particulate matter pollution sometimes demonstrate higher breast cancer rates. However, these correlations are complex due to multifactorial environmental exposures and socioeconomic variables influencing health outcomes. Emerging research is beginning to integrate environmental monitoring data with cancer registries to better understand these associations. Longitudinal studies focusing on populations residing near industrial zones or mining areas are necessary to elucidate the impact of chronic environmental iron exposure on breast cancer risk^[41]^.

#### Genetic susceptibility and iron overload syndromes in breast cancer

Genetic factors that regulate iron metabolism significantly influence individual susceptibility to iron overload and may play a pivotal role in breast cancer development. Among these, HH is the most extensively studied iron overload syndrome, caused predominantly by mutations in the *HFE* gene. These mutations disrupt iron homeostasis, leading to excessive iron absorption and systemic accumulation, which has been linked to increased cancer risk. HH is classically associated with heightened risks of liver cancer; however, emerging evidence suggests that iron accumulation may also contribute to breast carcinogenesis. Women with *HFE* mutations may experience altered iron metabolism that promotes oxidative DNA damage and inflammatory responses in breast tissue, thereby increasing the likelihood of malignant transformation. Although direct epidemiological data linking HH to breast cancer are limited, studies indicate that iron overload in breast epithelial cells can enhance estrogen metabolism dysfunction, a key factor in hormone-sensitive breast cancer development^[42,43]^.

Beyond *HFE* mutations, polymorphisms in genes involved in iron regulation – including those encoding ferroportin, transferrin receptors, ferritin, and hepcidin – may modulate iron burden and influence breast cancer susceptibility. Genetic variability affecting iron transport and storage can alter cellular oxidative stress levels, potentially increasing mutagenesis and tumor progression in mammary tissue. Furthermore, gene-environment interactions are critical; individuals genetically predisposed to iron overload may be more vulnerable to occupational or environmental iron exposures, compounding their risk of breast cancer. Recognizing the interplay between genetic susceptibility and iron overload syndromes in breast cancer is crucial for advancing personalized prevention and therapeutic strategies. Screening for iron metabolism-related genetic variants in high-risk populations, particularly among women with significant iron exposure, could facilitate early detection and intervention. Ultimately, integrating genetic insights with environmental risk assessments may improve breast cancer risk stratification and reduce disease burden^[44,45]^.

### Implications for public health and prevention

The evidence linking occupational and environmental iron exposure to breast cancer has important public health implications. Chronic or excessive iron exposure represents a potentially modifiable risk factor, particularly in populations with high occupational contact, environmental contamination, or dietary iron excess. Recognizing iron exposure as a contributor to breast carcinogenesis underscores the need for proactive preventive strategies and policy interventions^[46,47]^. Workplaces with high iron exposure, including mining, metal fabrication, welding, and smelting industries, should implement regular monitoring of airborne and particulate iron levels. Biomonitoring of exposed workers through serum ferritin, transferrin saturation, or other iron biomarkers can help identify individuals at elevated risk. Environmental surveillance of drinking water, soil, and industrial emissions in residential areas near iron-rich industrial zones is also essential to prevent chronic exposure^[48,49]^.

Preventive strategies should target both individual and population levels. Occupational interventions may include engineering controls, personal protective equipment, and rotation schedules to limit exposure. Public health initiatives can focus on minimizing environmental contamination and promoting safe dietary iron intake, especially in populations with high baseline exposure. Education and awareness campaigns are critical to inform workers, communities, and healthcare providers about the potential carcinogenic risks of chronic iron overload^[50,51]^. High-risk populations could benefit from tailored breast cancer screening programs. Incorporating iron exposure assessment into risk stratification frameworks may improve early detection and allow timely interventions. While further research is needed to establish standardized screening thresholds, combining exposure assessment with established risk factors (family history, hormonal profile) can enhance precision in identifying at-risk individuals^[52,53]^.

**Figure 1.**
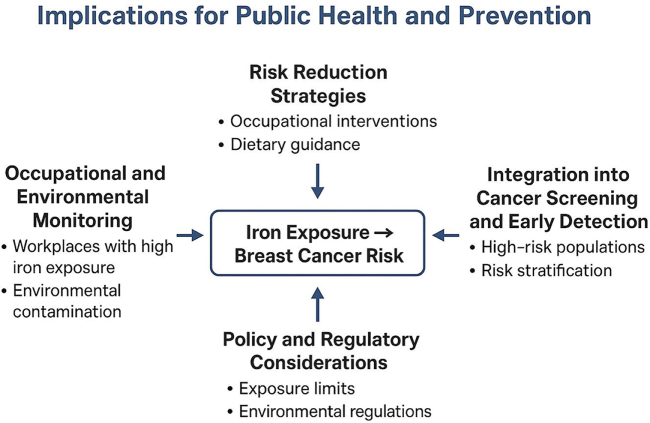
Implications for public health and prevention.

Regulatory frameworks should establish occupational exposure limits for iron and mandate compliance monitoring in industries with significant exposure risk. Environmental regulations should enforce limits on iron contamination in water and soil and provide remediation in affected areas. Collaborative approaches between occupational health agencies, environmental authorities, and cancer prevention programs are essential to ensure comprehensive risk reduction^[48]^. Ongoing epidemiological and mechanistic research is needed to clarify dose-response relationships, identify vulnerable subgroups, and evaluate the effectiveness of preventive interventions. Surveillance systems integrating environmental, occupational, and health data can facilitate early identification of high-risk populations and support evidence-based policy development (Fig. 1) ^[48,54]^.

## Conclusion

Occupational and environmental iron exposure represents an underrecognized but potentially significant risk factor in breast cancer etiology. Iron’s essential biological roles are counterbalanced by its capacity to induce oxidative stress, inflammation, and genotoxicity when present in excess. Disruptions in iron homeostasis – whether due to chronic exogenous exposure, genetic predisposition, or a combination of both – may contribute to breast carcinogenesis through multifaceted molecular pathways. While current epidemiological evidence linking iron exposure specifically to breast cancer remains limited, findings from related research and mechanistic studies underscore the plausibility of this association. Populations with occupational exposure to iron-containing dust and fumes, as well as communities residing near industrial pollution sources, warrant increased surveillance and protective measures. Moreover, genetic susceptibilities that influence iron metabolism may amplify individual risk, highlighting the need for integrated approaches combining environmental, occupational, and genetic factors in breast cancer prevention.
